# Comparative analysis of the complete mitogenomes of *Camellia sinensis* var. *sinensis* and *C. sinensis* var. *assamica* provide insights into evolution and phylogeny relationship

**DOI:** 10.3389/fpls.2024.1396389

**Published:** 2024-08-22

**Authors:** Li Li, Xiangru Li, Yun Liu, Junda Li, Xiaoyun Zhen, Yu Huang, Jianghua Ye, Li Fan

**Affiliations:** ^1^ College of Tea and Food Science, Wuyi University, Wuyishan, China; ^2^ College of Resources and Environment, Fujian Agriculture and Forestry University, Fuzhou, China

**Keywords:** *Camellia sinensis*, mitochondrial genome, genome comparison, codon preference, positive selection, phylogenetic analysis

## Abstract

**Introduction:**

Among cultivated tea plants (*Camellia sinensis*), only four mitogenomes for *C. sinensis* var. *assamica* (CSA) have been reported so far but none for *C. sinensis* var. *sinensis* (CSS). Here, two mitogenomes of CSS (CSSDHP and CSSRG) have been sequenced and assembled.

**Methods:**

Using a combination of Illumina and Nanopore data for the first time. Comparison between CSS and CSA mitogenomes revealed a huge heterogeneity.

**Results:**

The number of the repetitive sequences was proportional to the mitogenome size and the repetitive sequences dominated the intracellular gene transfer segments (accounting for 88.7%- 92.8% of the total length). Predictive RNA editing analysis revealed that there might be significant editing in NADH dehydrogenase subunit transcripts. Codon preference analysis showed a tendency to favor A/T bases and T was used more frequently at the third base of the codon. ENc plots analysis showed that the natural selection play an important role in shaping the codon usage bias, and Ka/Ks ratios analysis indicated *Nad1* and *Sdh3* genes may have undergone positive selection. Further, phylogenetic analysis shows that six *C. sinensis* clustered together, with the CSA and CSS forming two distinct branches, suggesting two different evolutionary pathway.

**Discussion:**

Altogether, this investigation provided an insight into evolution and phylogeny relationship of *C. sinensis* mitogenome, thereby enhancing comprehension of the evolutionary patterns within *C. sinensis* species.

## 1 Introduction

Tea is the oldest and most popular non-alcoholic soft drink in the world, with enormous economic, cultural and scientific value (Xia et al., 2017). The main class of cultivated tea plants (*Camellia sinensis*) consist of *C. sinensis* var. *sinensis* (L.) O. *Kuntze* (Chinary type), *C. sinensis* var. *assamica* (Masters) Chang (Assamica type) and *C. sinensis* var. *assamica* subssp. Lasiocalyx Planch (Cambodia type). Of which, *C. sinensis* var. *sinensis* (CSS) and *C. sinensis* var. *assamica* (CSA) have the most obvious distinction. CSS has small leaves and is mainly grown in China and some Southeast Asian countries, while CSA has large leaves and is widely grown in India and some hot countries except southern China (Mondal, 2014; Kumarihami et al., 2016). It has long been suggested that CSS and CSA may have different origins, and that CSA is composed of two distinct lineages (Chinese Assamica type and Indian Assamica type) that were domesticated independently (Meegahakumbura et al., 2016; Wambulwa, 2018). In recent years, several studies have investigated the genetic diversity and evolution between CSS and CSA by whole genomes resequencing or complete chloroplast genomes assembly (Yengkhom et al., 2019; An et al., 2020; Li et al., 2021a). These studies confirmed that after the tea plants went through the last glacial maximum of the Quaternary, the CSA began to diverge, and the more cold-resistant CSS started to emerge. Since then, the two have evolved in parallel, forming the existing classification groups of tea plants (Zhang et al., 2021, 2023). However, the dynamic evolution of mitochondrial genomes between CSS and CSA has never been assessed until now.

Plant mitochondria are a kind of semi-autonomous organelle in eukaryotic cells. As a source of ATP energy, plant mitochondria play a variety of cellular functions during plant growth and development (Klingenberg, 2008; Liberatore et al., 2016; Niu et al., 2022). Plant mitochondrial genome (mitogenome) possess many unique features and complex dynamic structure (Kozik et al., 2019; Li et al., 2023), such as extreme variation in genome size (Petersen et al., 2020), very sparse gene distribution (Wu et al., 2022), large number of non-coding sequences (Zandueta-Criado and Bock, 2004), rich repeat sequences (Yang et al., 2022), ability to intracellular gene transfer (IGT) (Li et al., 2022), highly conservative gene sequences, and a large number of RNA editing (Fang et al., 2021). These characteristics make the plant mitogenome an important tool for studying the classification and evolution of plants (Ammiraju et al., 2007; Chen et al., 2017; Van de Paer et al., 2018; Sibbald et al., 2021). Therefore, the investigation of mitogenome is not only important for understanding mitochondrial function and cell metabolism regulation, but also provides an important theoretical basis for the study of plant species evolution and genetic diversity.

At present, only four mitogenomes of tea plants have been reported, and they all belong to Assamica type tea (*C. sinensis* var. *assamica*, CSA), three of which belong to Chinese Assamica type tea and one belongs to Indian Assamica type tea (Zhang et al., 2019; Rawal et al., 2020; Li et al., 2023). More mitogenome information of *C. sinensis* were needed to carry out further research. In this study, two complete mitogenomes of Chinary type tea plants (*C. sinensis* var. *sinensis*, CSS), including *C. sinensis* var. *sinensis* cv. Dahongpao (CSSDHP) and *C. sinensis* var. *sinensis* cv. Rougui (CSSRG) were sequenced and assembled using a combination of Illumina and Nanopore sequencing techniques. Both tea plants are excellent cultivars of Wuyi rock tea (Synonym: *Thea bohea* L.) with a long history. Of which CSSDHP is famous for being considered one of the most expensive teas in the world, more valuable by weight than gold (Rose, 2010; Li et al., 2021b), and CSSRG also is one of the highest-ranking oolong teas with the intensity of a spicy and cinnamon-like odor and a mellow and heavy taste (Xu et al., 2022). In our previous studies, the complete chloroplast genomes of CSSDHP (Accession number: MT773374) and CSSRG (Accession number: MT773375) had been assembled and could be available in the NCBI database (Li et al., 2021b; Fan et al., 2022). Here, in addition to mitogenomes of two Chinary type teas sequencing and assembly, the mitogenome structure, intracellular sequence transfer and RNA editing events have been further analyzed and compared with mitochondrial sequences of Assamica type tea. This comparative analysis would provide a more comprehensive perspective on the complexity of the mitogenome of *C. sinensis* and shed light on the evolution and phylogeny relationship of *C. sinensis.*

## 2 Methods and materials

### 2.1 Plant material

The cultivars of *C. sinensis* var. *sinensis* cv. Dahongpao (CSSDHP) and *C. sinensis* var. *sinensis* cv. Rougui (CSSRG) were obtained from Wuyi Mountain, Fujian Province (27°43′42.46″N, 118°0′14.40″E) in China. Young leaves were collected, mitochondria were isolated from leaves by using density gradient centrifugation and digested with DNase I (Promega, Madison, USA) to eliminate genomic DNA contamination (JRan et al., 2010). DNA were extracted using the plant DNA extraction kits (TransGene, Beijing, China), and the final DNA quality was detected by a NanoDrop spectrophotometer (Thermo Scientific, Carls-bad, CA, USA). DNA samples were preserved at −80°C at the Key Laboratory of Tea germplasm Genetic Resources of Wuyi University.

### 2.2 Genome sequencing, assembly and annotation

To obtain the full-length mitogenome sequence, short-read (Illumina) and long-read (Nanopore) sequencing technologies were used. The short raw reads were checked with FastQC v0.12.1 and trimmed by Trimmomatic v0.36. The long raw reads were base-called by using Albacore v2.1.7 (mean_qscore > 7) with barcode demultiplexing, and converted to fasta format with Samtools Fasta (http://www.htslib.org/doc/samtools.html). Two strategies were used to assemble the mitogenome. In the first strategy, the short clean reads were *de novo* assembled with GetOrganelle v1.6.4, potential mitochondrial contigs were extracted by aligning against the mitochondrial protein-coding genes from plant mitogenome database (ftp://ftp.ncbi.nlm.nih.gov/refseq/release/mitochondrion/) with BLAST v2.8.1+ (Dierckxsens et al., 2017). Then, the putative long mitochondrial reads were baited by mapping the Nanopore long reads to the potential mitochondrial contigs using BLASR v5.1 and assembled by Canu v2.1.1. In the second strategy, all Nanopore long reads were assembled *de novo* by using Canu directly (Koren et al., 2017). Subsequently, BWA were used to map the short clean reads to the draft contigs and improved the draft contigs with Pilon v1.22. Then, Bandage (Wick et al., 2015) was used to check whether these contigs were circular. Finally, the corrected contigs obtained from the above two assembly strategies were aligned with each other using MUMmer v3.23, and the result showed that these two contigs were identical. Examine the aligned bam files using IGV to verify the results of the assembly (Robinson et al., 2011). Based on the above assembly steps, two mitogenomes, CSSDHP and CSSRG were obtained.

Protein-coding genes and Ribosomal RNA (rRNA) were annotated by their similarity to published plant mitochondrial sequences and by using BLAST searches. The tRNA genes were annotated using tRNAscanSE (http://lowelab.ucsc.edu/tRNAscan-SE/) (Jackman et al., 2020). The position of each coding gene was determined using BLAST searches against reference mitogenome genes (OL989850 and OM809792). ORFs were predicted by ORF Finder (https://www.ncbi.nlm.nih.gov/orffinder/) with the standard genetic code and a minimal length of 102 nt, and ORFs longer than 300 bp were annotated by Blast2GO with default parameters (Conesa et al., 2005). Manual corrections of genes for start/stop codons and for intron/exon boundaries were performed in SnapGene Viewer by referencing the reference mitogenomes (OL989850 and OM809792). The mitogenome maps were drawn using the OGDRAW tool (Greiner et al., 2019).

These two complete mitogenome sequences and accompanying gene annotations had been deposited in the NCBI GenBank (Accession numbers: PP212895, CSSDHP; PP212896, CSSRG). Four reported *C. sinensis* mitogenomic data, including CSAOL (Accession number: OL989850), CSAOM (Accession number: OM809792), CSAMK (Accession number: MK574876 and MK574877, assembled into two rings) and CSAMH (Accession number: MH376284), was downloaded from the NCBI website. Incorrect annotation information was checked and corrected.

### 2.3 Mitogenome synteny analysis

The BLASTN program was used for pairwise comparison of six mitogenomes of *C. sinensis*. Interspecies homologous regions were searched to indicate mitogenomic synteny among six *C.sinensis* species, fragments shorter than 100 bp was excluded from the analysis. Homologous sequences with a length of more than 500 bp were retained as conserved collinear blocks and then were collinearly visualized using AliTV 1.0.6 (https://alitvteam.github.io/AliTV/d3/AliTV.html) (Zhang et al., 2013).

### 2.4 Repetitive sequence analysis

The dispersed long sequence repeat across the mitogenome (Forward, Palindromic, Reverse and Complement) was detected by REPuter program (https://bibiserv.cebitec.uni-bielefeld.de/reputer/) with the minimum repeating size set to 30 and the hamming distance set to 3 (Kurtz et al., 2001). Simple sequence repeats (SSRs) were detected by MISA program (https://webblast.ipk-gatersleben.de/misa/) with default parameters (Beier et al., 2017).

### 2.5 Intracellular gene transfer

Homologous DNA fragments were discovered between the chloroplast genome and mitogenome by BLASTN (ncbi-blast-2.2.30+) with 70% identity as the threshold and the e-value of 1e^-6^ (Chen et al., 2015). To eliminate redundant detection, only a single IR (Inverted repeat) region of the chloroplast genome were used for analysis, and fragments with overlapping locations were combined into unique fragments. The reported chloroplast genomes of CSSDHP, CSSRG, CSAOL and CSAMK was downloaded from NCBI with accession numbers MT773374 and MT773375, OL450397and MH019307, respectively.

### 2.6 RNA editing prediction, codon usage analysis and Ka/Ks evaluation

RNA editing events were predicted based on the online website PREPACT v3.12.0 (http://www.prepact.de/) (Lenz et al., 2018), and the setting standard are: cutoff value = 0.001. To avoid data bias, the protein-coding genes with lengths < 300 bp were excluded in codon usage calculations (Rosenberg et al., 2003). The relative synonymous codon usage (RSCU) values, the effective number of codons (ENc) value and the contents of GC at the first, second and third positions of each codon of each gene were evaluated by cusp (EMBOSS v6.6.0.0) (http://emboss.toulouse.inra.fr/cgi-bin/emboss/help/cusp). RSCU = 1 indicated that there was no preference for the use of this codon, and RSCU > 1 indicates that the codon was used preferentially by amino acids, while if RSCU < 1, the codon usage is contrary. Parity rule 2 (PR2) plot analysis were also conducted to investigate codon usage bias based on a plot of AT-bias [A3/(A3 + T3)] and GC-bias [G3/(G3 + C3)] at the third codon position of the four-codon amino acids in entire genes (Huang et al., 2022). The ENc-plot of ENc values plotted against GC3s values (ENc *vs* GC3s) was used to analyze the influence of base composition on the codon usage in a genome. If the points lie on or around the standard curve, the codon usage was constrained only by mutation bias. Otherwise, the codon usage pattern is influenced by other factors, such as natural selection (Wright, 1990). Ka/Ks value for each gene was calculated using the KaKs_Calculator 2.0. Ka/Ks >1 means positive selection, Ka/Ks =1 mean neutral selection, and Ka/Ks <1 mean negative selection (Wang et al., 2010). The heatmap of Ka/Ks values was drawn using ChiPlot (https://www.chiplot.online/), with a clustering method of complete and euclidean distance.

### 2.7 Phylogenetic analysis

A total of 20 plant mitogenomes were downloaded from NCBI and were used to construct phylogenetic trees (**Supplementary Table S10**). The mitogenomes of *Zea mays*, *Sorghum bicolor*, *Triticum aestivum*, and *Ginkgo biloba* were employed as outgroups. First, MUMmer v3.23 and BLAT software were used for global and local alignment between the sample sequence and the reference genome (CSSDHP) under default parameters, and the alignment was trimmed by Gblocks_0.91b to remove low-quality regions with the parameters: -t=d -b4 = 5 -b5=h. A total of 573 SNPs was found. For each mitogenome, all SNPs were connected in the same order to obtain sequences of the same length for the construction of phylogenetic trees. Then, the genome-wide phylogenetic trees were constructed by both Maximum-likelihood (ML) and Bayesian inference (BI) methods.

The ML method was performed using PhyML v3.0 (Guindon et al., 2010). Nucleotide substitution model selection was estimated with jModelTest 2.1.10 and the Smart Model Selection feature in PhyML v3.0. The model GTR+I+G was selected for ML analyses with 1,000 bootstrap replicates for calculating the bootstrap values (BS) of the topology. MrBayes v3.2.6 (Ronquist et al., 2012) was used in BI analysis. Four chains (three heated and one cold) and two runs of 2 million generations were carried out, with each run being sampled every 100 generations. The first 10% of samples was discarded as burn-in, and the remaining trees were used to estimate Bayesian posterior probabilities (BPPs). The phylogenetic tree was visualized with iTOL v6 (Letunic and Bork, 2021).

## 3 Results

### 3.1 Mitogenome assembly and annotation

**Figure 1:**
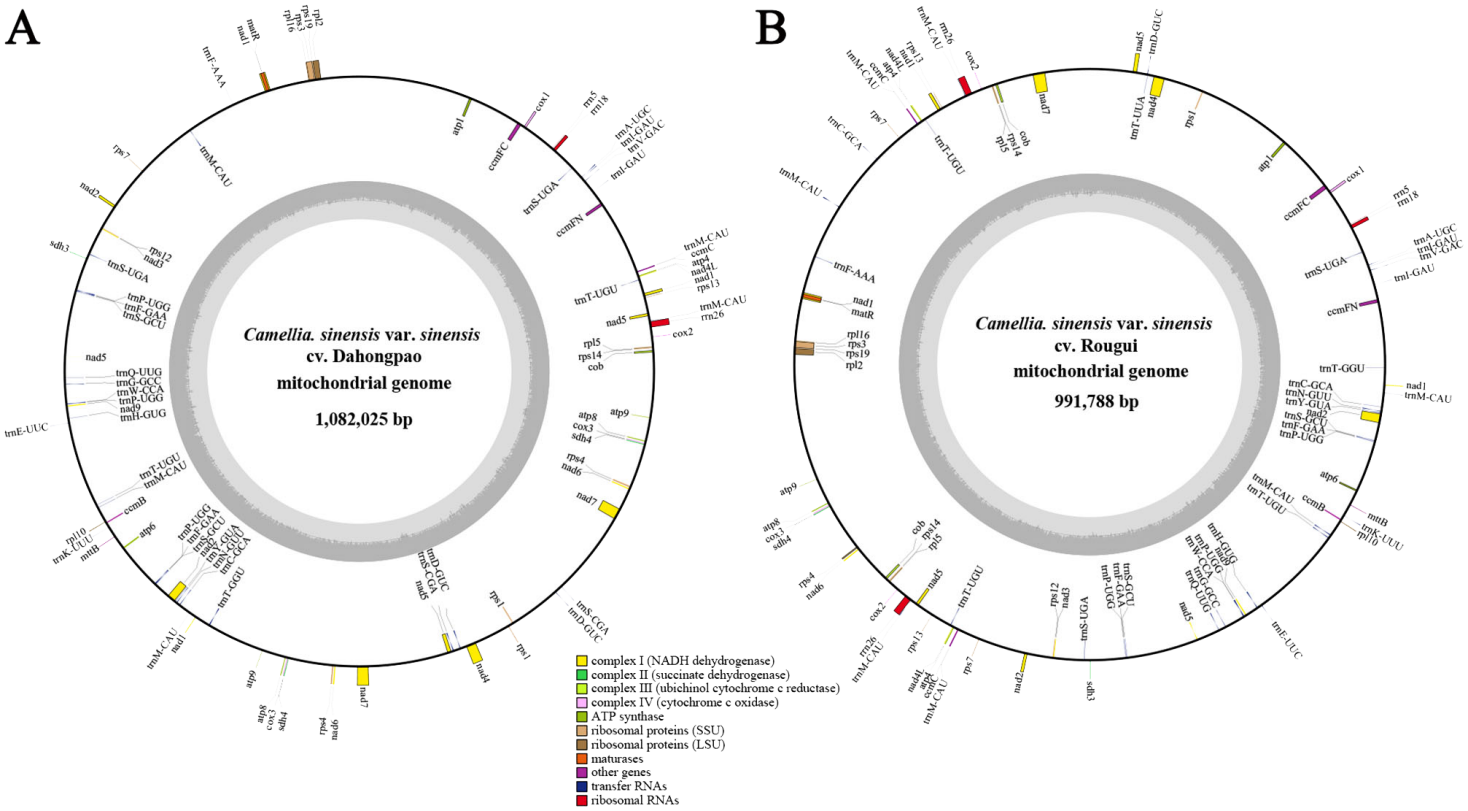
*C. sinensis* var. *sinensis* cv. Dahongpao **(A)** and *C. sinensis* var. *sinensis* cv. Rougui **(B)** mitochondrial genome circular map.

The complete circular mitogenomes of *C. sinensis* var. *sinensis* cv. Dahongpao (CSSDHP) and *C. sinensis* var. *sinensis* cv. Rougui (CSSRG) were obtained using a combination of Illumina and Nanopore data. A total of 60,413,998 Illumina reads (about 9.1 Gb, average read length 150 bp) and 1,008,363 Nanopore reads (about 5.1 Gb, average read length 5,032 bp) were mapped to the complete mitogenome of CSSDHP. The average coverage reached 8,361× and 4,689× sequencing depth. A total of 55,973,904 Illumina reads (about 8.4 Gb, average read length 150 bp) and 652,559 Nanopore reads (about 4.9 Gb, average read length 7,608 bp) were mapped to the complete mitogenome of CSSRG. The average coverage, with 45.41× for CSSDHP and 87.61× for CSSRG, was 99.95% and 100%, respectively (**Supplementary Tables S1**; **Supplementary Figures S1**, **S2**). The assembly using error-corrected Nanopore reads resulted in circular genomes of 1,082,025 bp for CSSDHP and 991,788 bp for CSSRG. A total of 79 genes were identified in the mitogenome of CSSDHP, including 46 protein-coding genes (PCGs), 30 tRNA genes, and 3 rRNA genes. A total of 87 genes were identified in the mitogenome of CSSRG, including 47 PCGs, 37 tRNA genes, and 3 rRNA genes (**Figure 1**). When compared with the four reported *C. sinensis* mitogenomes, the GC content of all six mitogenomes was between 45% and 46%. However, the annotated genes and the number of genes differed significantly across the six mitogenomes (**Table 1**).

**Table 1:** General features of six *C. sinensis* mitogenomes.

Genome Features	*C. sinensis* var. *sinensis* (Chinary type)		*C. sinensis* var. *assamica* (Assamica type)			
			Chinese Assamica type			Indian Assamica type
Species code	CSSDHP	CSSRG	CSAOL	CSAOM	CSAMK	CSAMH
NCBI serial number	PP212895	PP212896	OL989850	OM809792	MK574876 and MK574877	MH376284
Location of sample	Fujian, China	Fujian, China	Hunan, China	Hunan, China	Yunnan, China	Assam, India
Genome size (bp)	1082025	991788	1081966	914855	879048	707441
Number of PCGs	46	46	47	42	40	31
Number of tRNA genes	35	37	30	30	23	23
Number of rRNA genes	3	4	4	3	3	3
GC content (%)	45.68	45.72	45.62	45.66	45.67	45.75

CSSDHP, CSSRG, CSAOL, CSAOM and CSAMK have the complete mitogenomes while CSAMH is missing seven PCGs. A total of 46 (CSSDHP), 46 (CSSRG), 47 (CSAOL), 42 (CSAOM), 40 (CSAMK) and 31 (CSAMH) PCGs were annotated in six *C. sinensis* mitogenomes, respectively. *Atp8*, *Atp9*, *Nad6*, *Nad7*, *Cox3*, *Rps1*, *Rps4* and *Sdh4* were replicated in CSSDHP mitogenome, *Atp9*, *Rpl2*, *Rpl16*, *Rps3* and *Rps19* were replicated in CSSRG mitogenome, *Atp9* (×3), *Rpl2*, *Rpl16*, *Rps3* (×3) and *Rps19* (×4) were replicated in CSAOL mitogenome, *Atp4*, *Nad4L*, CcmC and *Rps19* were replicated in CSAOM mitogenome, *Sdh3* and *Rps19* were replicated in CSAMK mitogenome, and *Rps19* were replicated in CSAMH mitogenome (**Table 1**; **Supplementary Table S2**). In addition, *trnM-CAT(x5)*, *trnT-TGT*, *trnI-GAT*, *trnS-TGA*, *trnP-TGG*(x3), *trnF-GAA*, *trnS-GCT*, *trnS-CGA* and *trnD-GTC* were replicated in CSSDHP mitogenome, *Rna26*, *trnM-CAT*(x7), *trnT-TGT* (x3), *trnI-GAT*, *trnS-TGA*, *trnP-TGG*(x3), *trnF-GAA*, *trnS-GCT* and *trnC-GCA* were replicated in CSSRG mitogenome, *Rrna18*, *ttrnM-CAT*(x4), *trnI-GAT*, *trnS-TGA*(x3), *trnP-TGG*, *trnS-GCT*, *trnN-GTT* and *trnC-GCA* were replicated in CSAOL mitogenome, *trnM-CAT* (x5), *trnI-GAT*, *trnS-TGA*, *trnP-TGG*, *trnS-GCT* and *trnC-GCA* were replicated in CSAOM mitogenome, *trnM-CAT* (x4), *trnP-TGG* (x3), *trnF-GAA*, *trnE-TTC*, *trnY-GTA* and *trnC-GCA* were replicated in CSAMK mitogenome, and *trnM-CAT*(x4), *trnI-GAT*, *trnS-TGA* (x3), *trnS-GCT* and *trnN-GTT* were replicated in CSAMH mitogenome. In six *C. sinensis* mitogenomes, *Nad4L*, *CcmFC*, *Rpl2*, *Rps3*, *trnT-TGT*, *trnI-GAT*, *trnA-TGC*, *trnS-TGA* and *trnT-GGT* has one intron, *Nad4* has two introns, *Nad1*, *Nad2*, *Nad5* and *Nad7* have four introns. One of the two *Sdh3s* in CSAMK mitogenome has one intron (**Supplementary Table S2**).

### 3.2 Mitogenome collinearity

**Figure 2:**
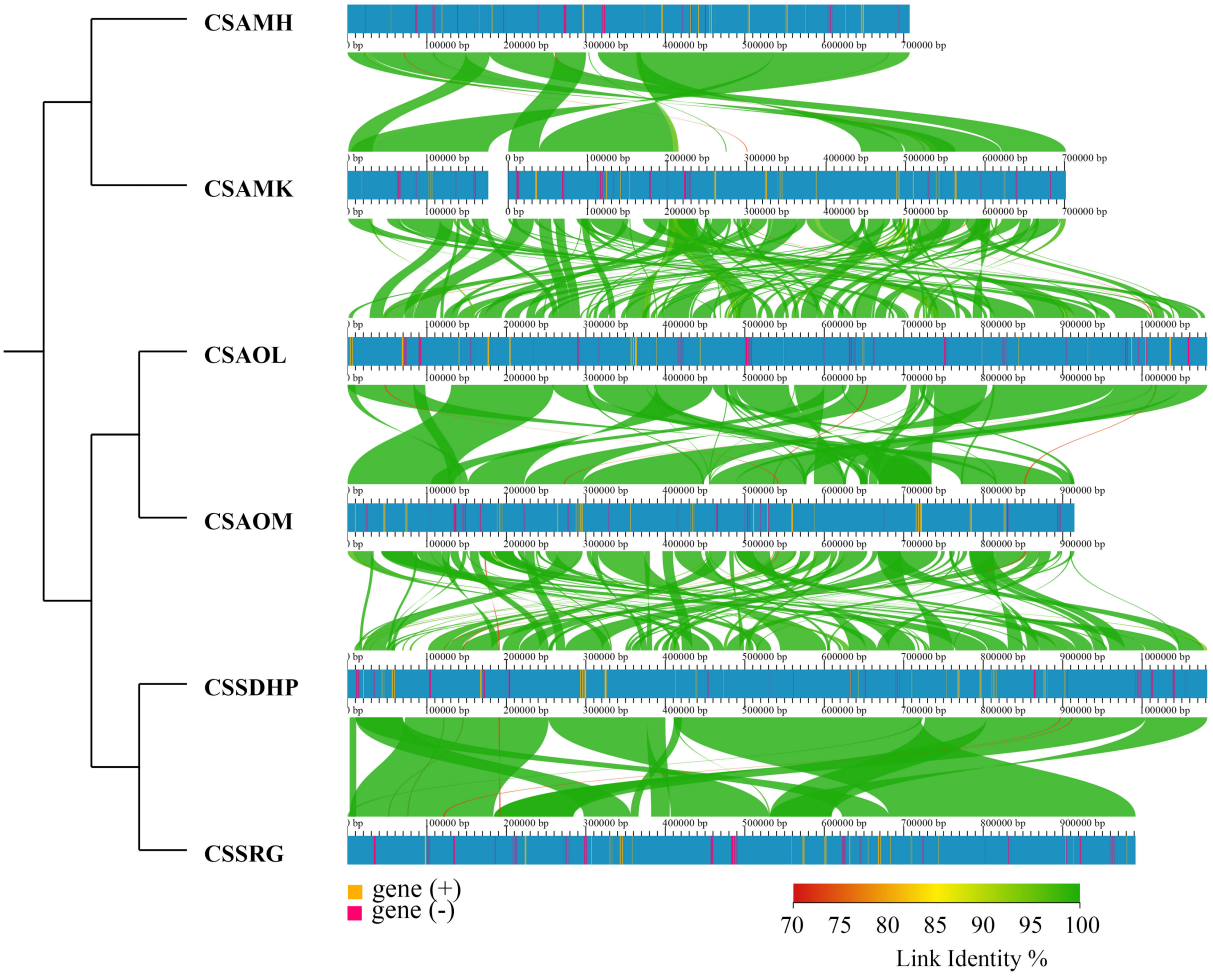
Collinear analysis of 6 *C. sinensis* species.

To assess the relationship between the six mitogenomes of *C. sinensis* species, the BLASTN program was performed to compare homologous genes and their sequence arrangement. The conserved collinearity blocks that were over 500 bp in length were identified for analysis. The results showed that while many homologous collinear blocks were detected in the six mitogenomes of *C. sinensis* species, the order of arrangement of collinear blocks among six mitogenomes exhibited inconsistency. The collinear cluster analysis showed that six *C. sinensis* mitogenomes were classified according to the degree of collinearity. CSSDHP and CSSRG, both from Fujian Province, China, are clustered together; CSAOL and CSAOM, both from Hunan Province, China, are clustered together; and CSAMK from Yunnan Province, China, are clustered together with CSAMH from Assam, India (**Figure 2**).

### 3.3 Repetitive sequences analysis

**Figure 3:**
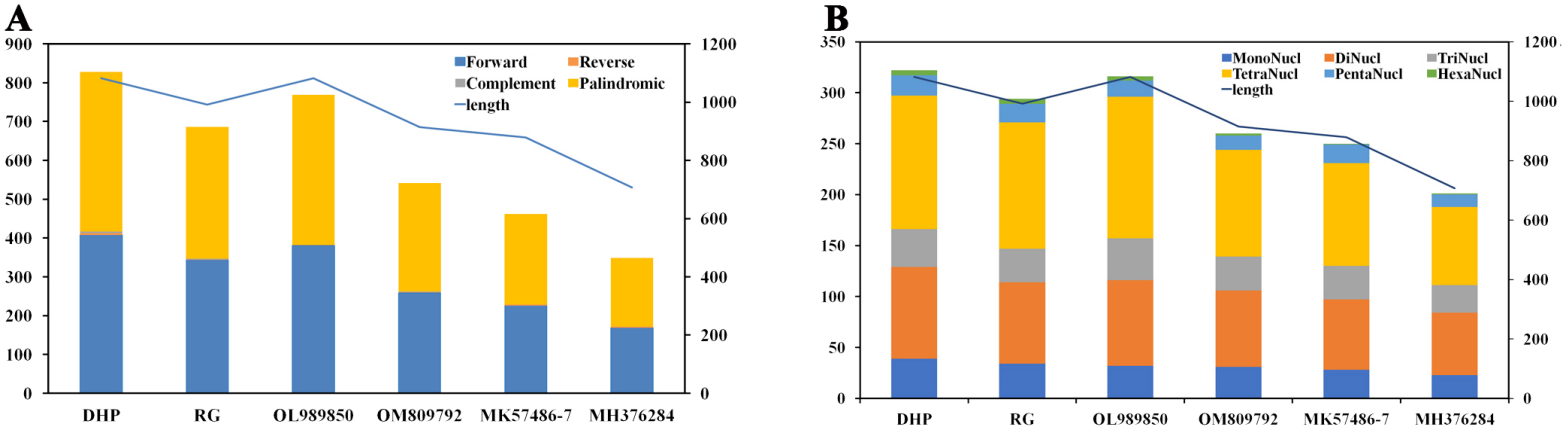
*C. sinensis* mitochondrial repeats. **(A)** The dispersed long sequence repeats. **(B)** The simple sequence repeats. Histograms display the repeat number of given lengths and the curve show the size of the mitochondrial genome.

A large number of repetitive sequences were found in the mitogenome of tea plant. There were 828 pairs of dispersed long repeats with a length ≥ 30 bp in the CSSDHP mitogenome, including 408 pairs of forward repeats, 411 pairs of palindromic repeats, 5 pair of reverse repeats and 4 pair of complementary repeats. There were 686 pairs of dispersed long repeats with a length ≥ 30 bp in the CSSRG mitogenome, including 344 pairs of forward repeats, 339 pairs of palindromic repeats, 1 pairs of reverse repeats and 2 pairs of complementary repeats. When compared with the four reported CSA species, the number of long repeats was 828 (CSSDHP), 686 (CSSRG), 769 (CSSOL), 542 (CSSOM), 462 (CSSMK) and 349 (CSSMH), respectively. The number of the long repetitive sequences was proportional to the total length of the mitogenome (**Supplementary Table S3**; **Figure 3A**).

A total of 322 and 294 SSRs were detected in CSSDHP and CSSRG mitogenomes, respectively. The number of SSRs in four reported CSA mitogenomes was 316 (CSAOL), 260 (CSAOM), 250 (CSAMK) and 201 (CSAMH), respectively. SSR tetra-nucleotide was the most abundant SSR type. The number of SSRs was proportional to the total length of the mitogenome (**Supplementary Table S4**; **Figure 3B**).

### 3.4 IGTs between organellar genomes

**Figure 4:**
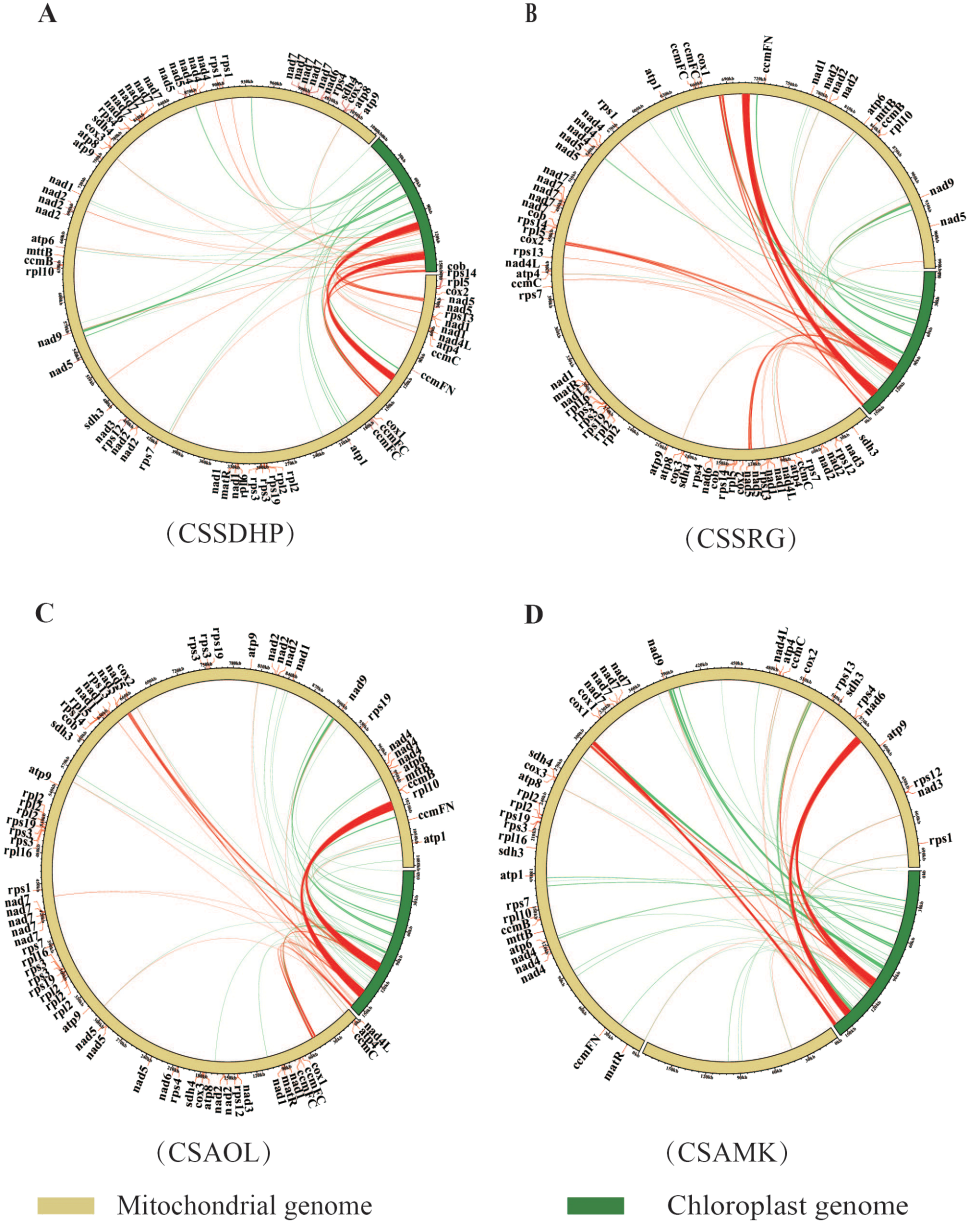
Schematic for the chloroplast-to-mitochondrial gene transfer. Sequence similarity between the mitochondrial and chloroplast genomes in **(A)** CSSDHP, **(B)** CSSRG, **(C)** CSAOL and **(D)** CSAMK. Homologous sequences connected by red lines are repetitive sequences, and non-repetitive sequences are connected by green lines.

The global alignment between the chloroplast genome and mitogenome for each of 4 C*. sinensis* species showed a total of 43 (20,733 bp), 46 (21,607 bp), 40 (21,701 bp) and 46 (21,496 bp) mitogenomic DNA fragments homologous to the chloroplast genome, accounting for 1.91%, 2.18%, 2.01% and 2.45% of the length of the whole mitogenome in CSSDHP, CSSRG, CSAOL and CSAMK, respectively (**Figure 4**). The majority of homologous fragments for each species occurred in the size range of 100 - 500 bp. It was noticed that most homologous regions involved repeat sequences, including SSR sequences or Long repeat sequences (especially the IR region), which account for 90.4% (18,746 bp, CSSDHP), 91.9% (19,849 bp, CSSRG), 92.8% (20,136 bp, CSAOL), and 88.7% (19,058 bp, CSAMK) of the total homologous sequence length, respectively. Among the four mitogenomes, the longest segments homologous to the chloroplast genome were 7,722 bp (CSSDHP), 7,721 bp (CSSRG), 9572 bp (CSAOL), and 6661 bp (CSAMK), all of which were in the IR region of their respective chloroplast genomes. In addition, within these homologous mitogenomic DNA, there were ten complete genes, all of which are tRNA genes (*trnH-GUG*, *trnD-GUC*, *trnM-CAU*, *trnW-CCA trnP-UGG*, *trnI-CAU*, *trnI-GAU*, *trnA-UGC*, *trnV-GAC* and *trnN-GUU*) (**Supplementary Table S5**).

### 3.5 Prediction of RNA editing

**Figure 5:**
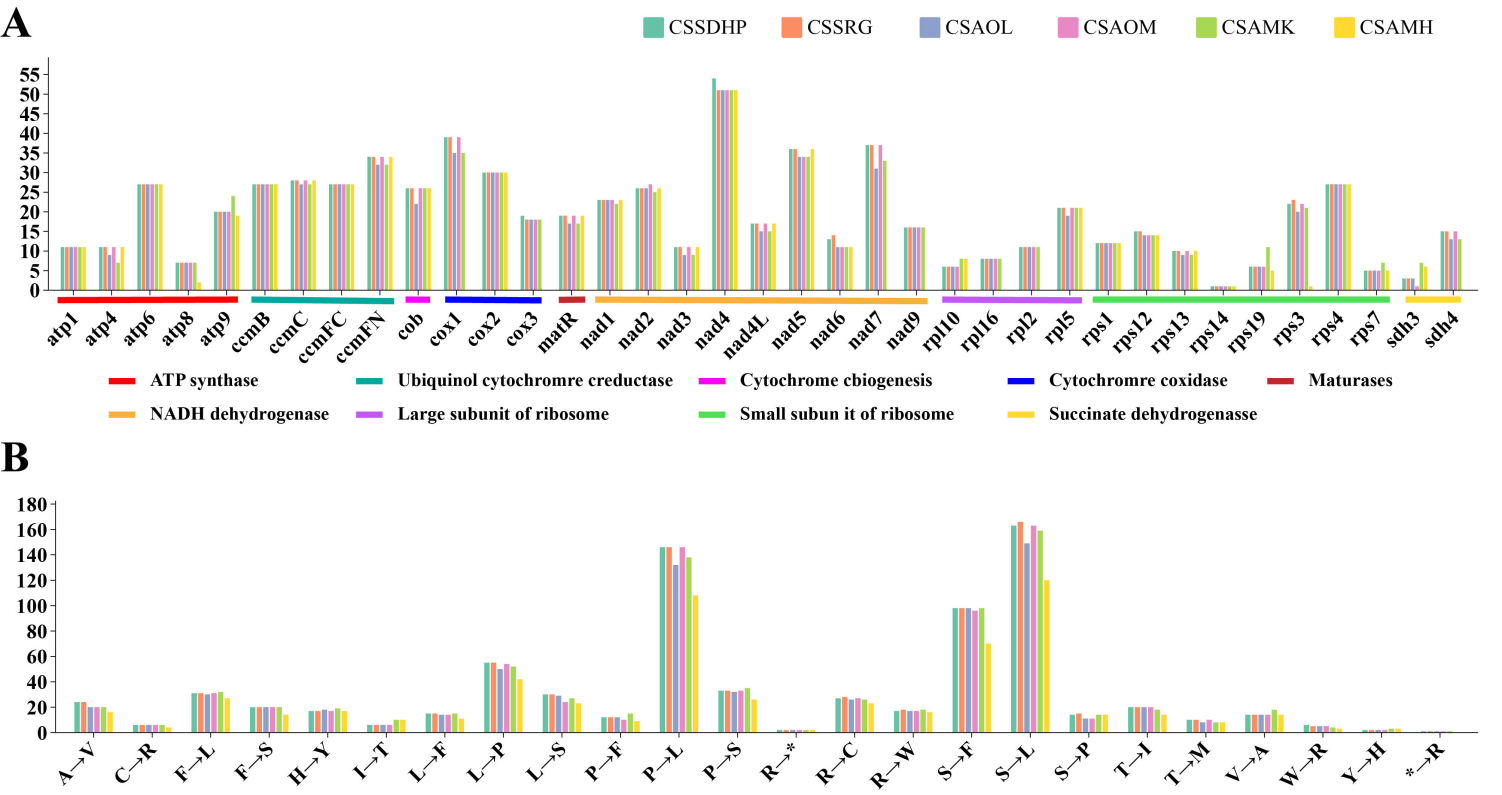
The Prediction of RNA Editing in six *C. sinensis* mitogenomes. **(A)** RNA editing sites in different coding genes. **(B)** Amino acid conversion type.

**Figure 6:**
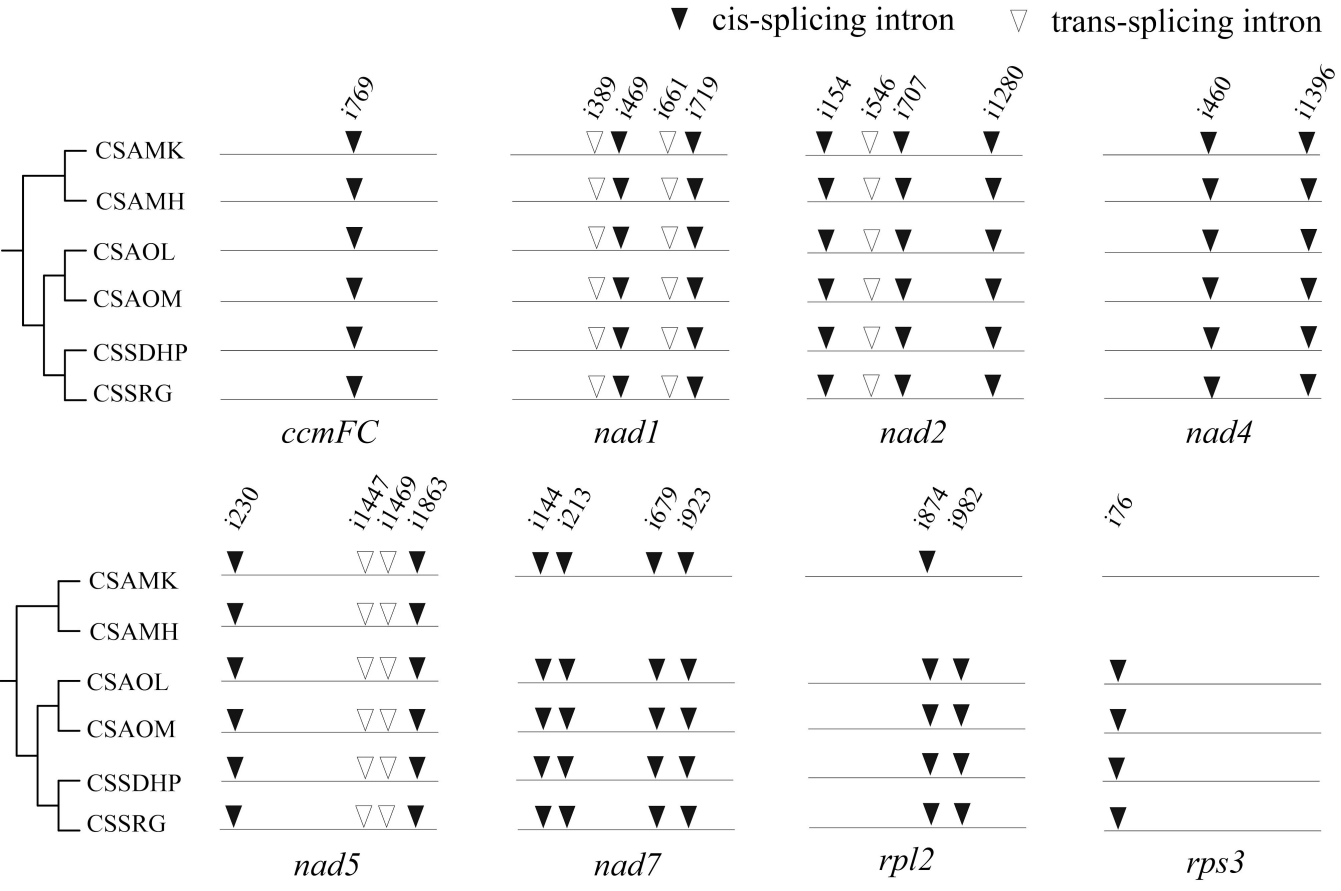
Comparison of mitochondrial introns among six *C. sinensis*. The arrowhead indicates the position of an intron insertion. Solid and hollow triangles represent cis- and trans-spliced introns, respectively.

RNA editing site were predicted in six mitogenomes of tea plant (**Supplementary Table S6**). There were a total of 720 (CSSDHP), 718 (CSSRG), 679 (CSAOL), 710 (CSAOM), 701 (CSAMK), and 546 (CSAMH) RNA editing sites identified in 37 gene types, respectively. The results showed that there was not only U-to-C RNA editing types, but also C-to-U RNA editing types. Among them, the *Nad4* gene had the most predictive RNA editing sites (51-54 sites), followed by *Nad5* (34-36 sites), while *Rps14* had minor editing sites (only 1 site) (**Figure 5A**). Among these substitutions, the most amino acid changes were serine to leucine (S-to-L), proline to leucine (P-to-L), and serine to phenylalanine (S-to-F), while stop codon to Arginine (*-to-R) were the least (**Figure 5B**). Most amino acid changes involved the conversion of amino acid hydrophobicity, 7.0% of the RNA editing amino acids were converted from hydrophobic to hydrophobic, 17.1% from hydrophobic to hydrophilic, 2.8% from hydrophilic to hydrophilic, 72.7% from hydrophilic to hydrophobic, and only 4% involved the conversion of termination codons. In each of the six mitogenomes, an editing event from CGA (Arginine) to UGA (Termination codon) were predicted in *CcmFC* and *Atp9* genes. In addition to CSAMH, an editing event from UGA (Termination codon) to CGA (Arginine) was predicted in *Rps19* gene. In addition to CSAOL,CSAMK, and CSAMH, two editing events from ACG (Threonine) to ATG (Initiation codon) were predicted in *Cob*, *Cox1*, *Nad5*, *Nad4L* and *Cox2* genes (**Supplementary Table S6**). In addition, *Nad1*, *Nad2*, and *Nad5* genes contained both cis-spliced and trans-spliced introns, while the *CcmFC*, *Nad4*, *Nad7*, *Rpl2*, and *Rps3* genes contained only cis-spliced introns (**Figure 6**).

### 3.6 Codon preference

**Figure 7:**
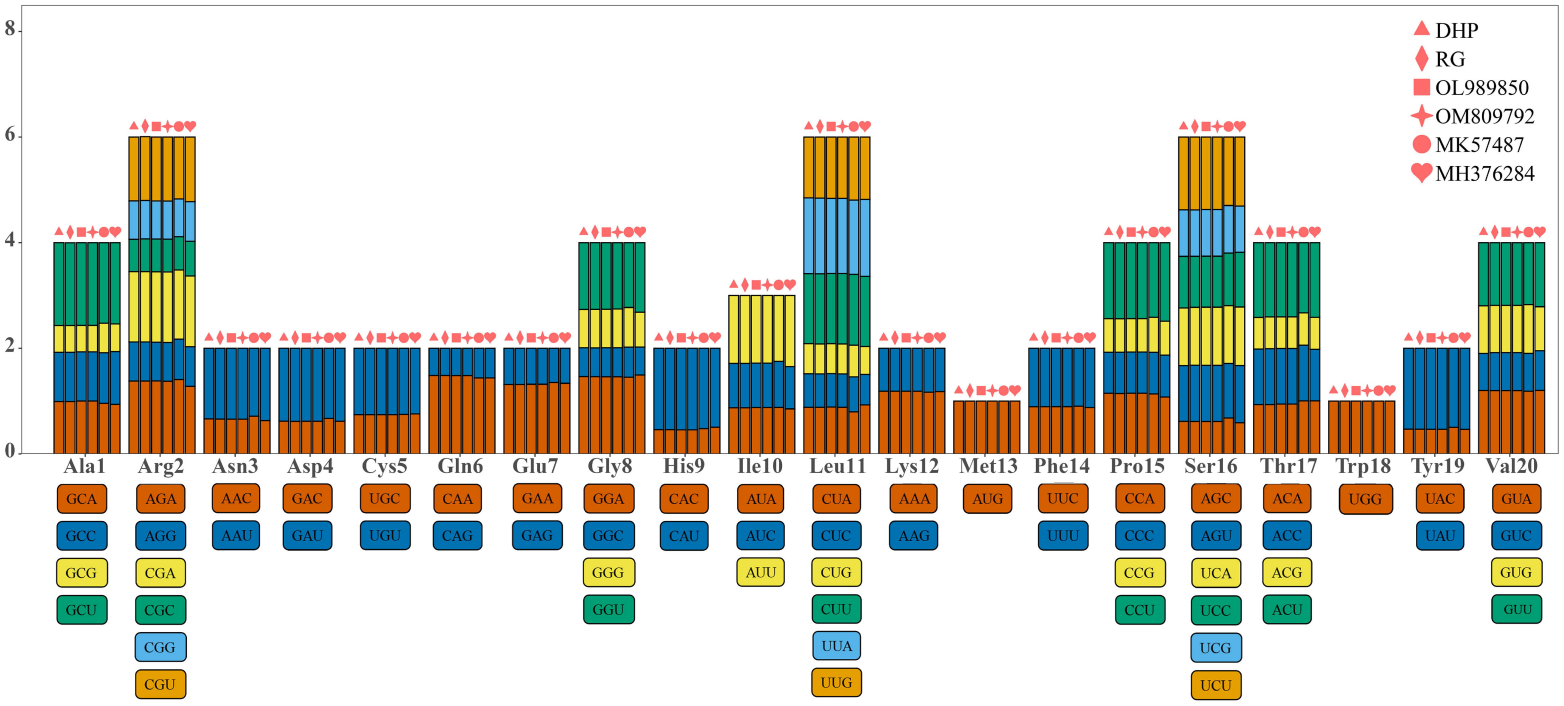
Relative synonymous codon usage (RSCU) of six *C. sinensis* mitogenomes.

The patterns of synonymous codon usage and the preference for G/C-ended codons were analyzed by RSCU analysis of codons. The results showed that most amino acids except methionine (AUG) and tryptophan (UGG) had bias in codon usage pattern (**Figure 7**). In the six mitogenomes, Alanine (Ala) had a high preference for the use of GCU, and its average RSCU value was the highest among mitogenome PCGs (RSCU: 1.5249—1.567), followed by Histidine (His) for the use of CAU (RSCU: 1.4948—1.5448), the termination codon for the use of UAA (RSCU: 1.4545—1.5429) and Tyrosine (Tyr) for the use of UAU (RSCU: 1.4961—1.5342). Tyrosine had the fewest preference for the use of UAC, with only 0.4647— 0.5039. Except for UUG, all codons with RSCU>1 end in A/T (**Supplementary Table S7**).

**Figure 8:**
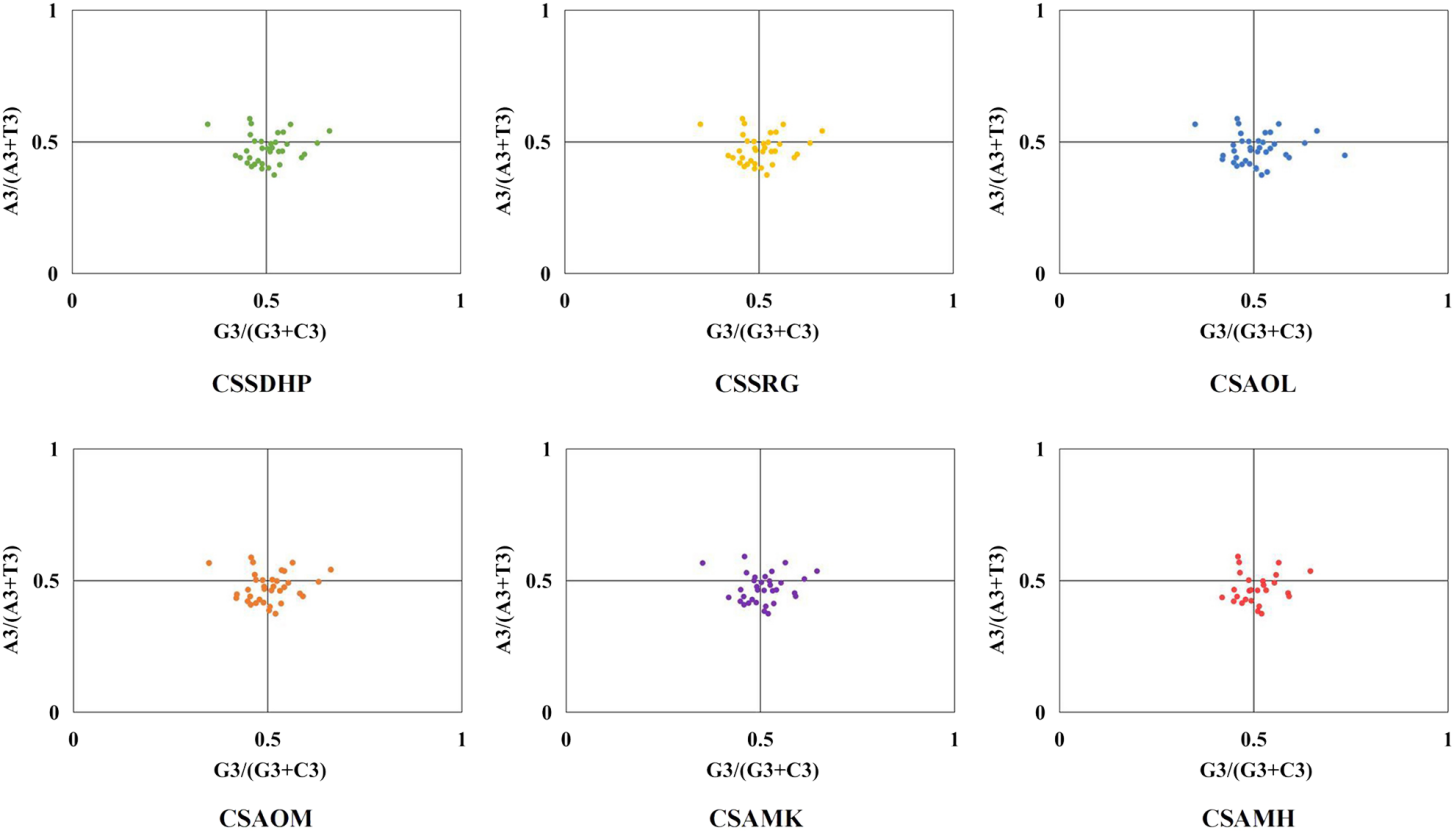
PR2-plot of six *C. sinensis* mitogenomes.

**Figure 9:**
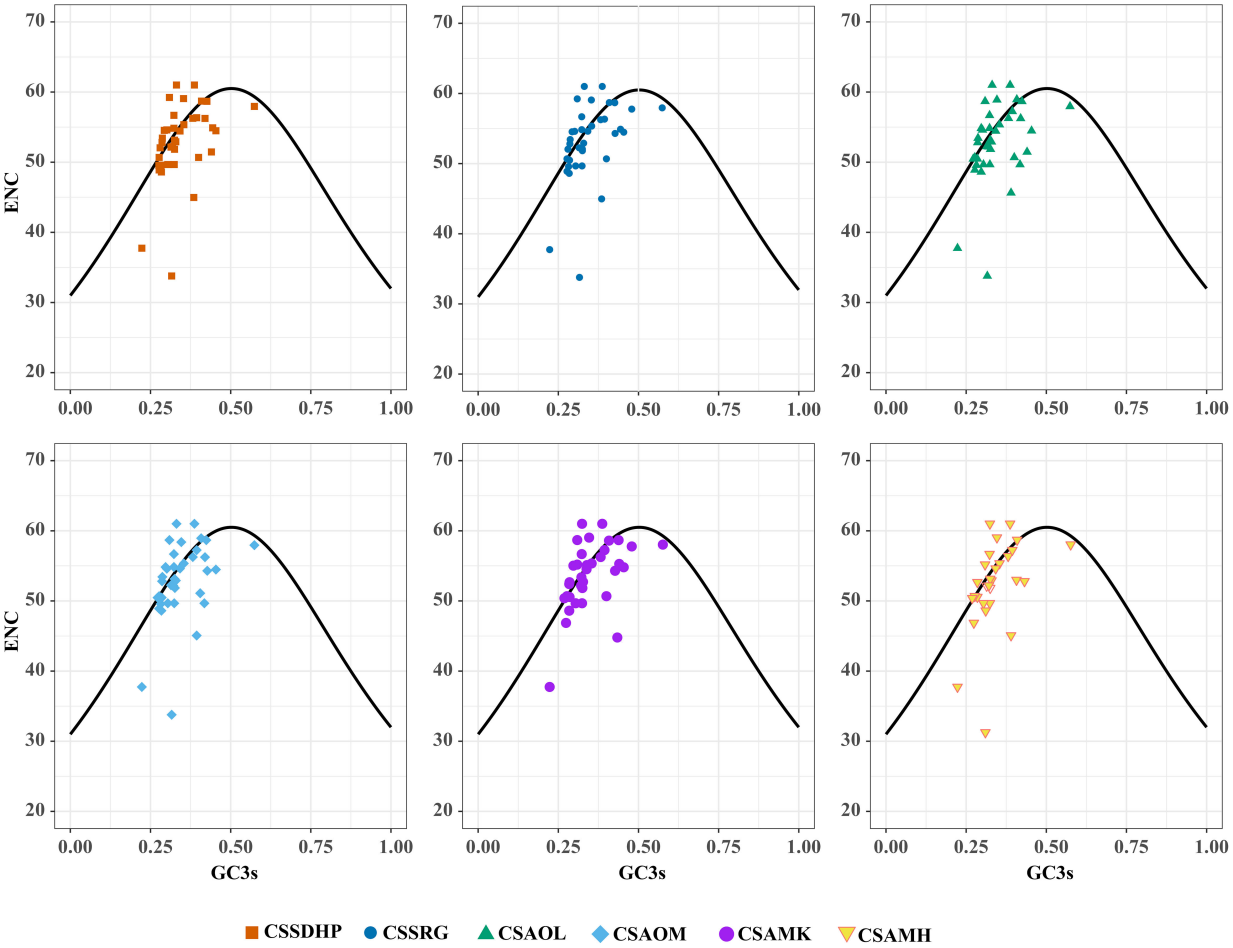
ENc-plot of six *C. sinensis* mitogenomes.

The GC content was calculated for the first (GC1), second (GC2), and third (GC3) positions of the PCGs in 6 mitogenomes and results showed average GC content of these different positions (GC1, GC2, and GC3) were less than 50% (**Supplementary Table S8**), suggesting an AT bias. PR2 plot analysis was further conducted to assess the codon usage bias. In all six mitogenomws, most genes were distributed in the lower quadrant of the PR2-plot (**Figure 8**), implying that T (pyrimidines) were used more frequently than A (purines) in *C. sinensis* codons. ENc-plot analysis (ENc *vs* GC3S) was used to determine the major factors affecting codon usage bias, and the result showed only a few points lay near the curve, however, most of the genes with lower ENc values than expected values lay below the curve (**Figure 9**), suggesting the codon usage bias was slightly affected by the mutation pressure, but selection pressure and other factors have played an important role.

### 3.7 Ka/Ks ratio analysis

**Figure 10:**
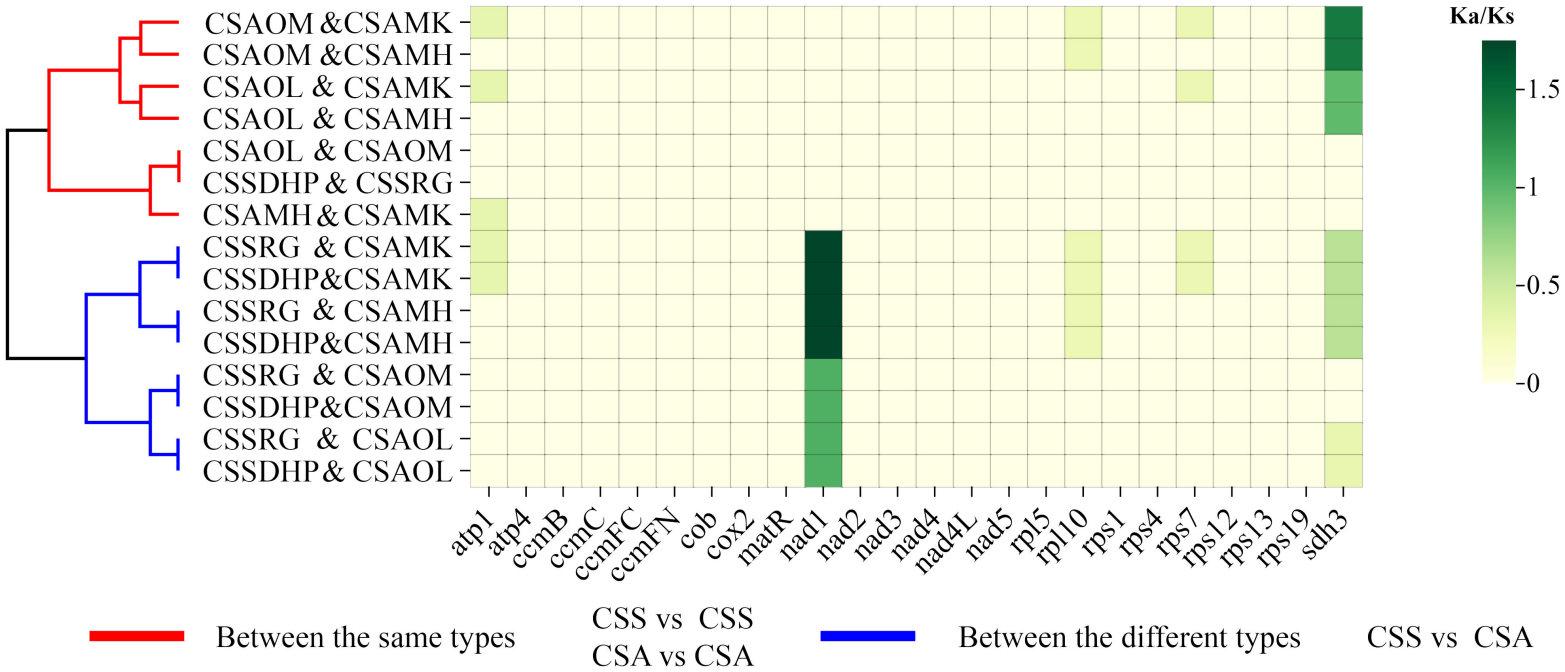
Analysis of Ka/Ks substitution rates among six *C. sinensis* mitogenomes.

The PCGs of six mitogenomes were compared in pairs, and the Ka/Ks ratios were calculated for the shared PCGs between the pairs. The results showed that the Ka/Ks ratio of most genes were less than 0.5 or equal to 0, suggesting that those genes had undergone significant purification selection. In contrast, Ka/Ks ratios of two genes (*Nad1* and *Sdh3* gene) were greater than 1, indicating positive selection of these two genes in *C. sinensis* species. In addition, Ka/Ks ratios between two same type species (CSS *vs* CSS or CSA *vs* CSA) and two different type species (CSS *vs* CSA) were clearly divided into two clusters (**Figure 10**, **Supplementary Table S9**).

### 3.8 Phylogenetic analysis

**Figure 11:**
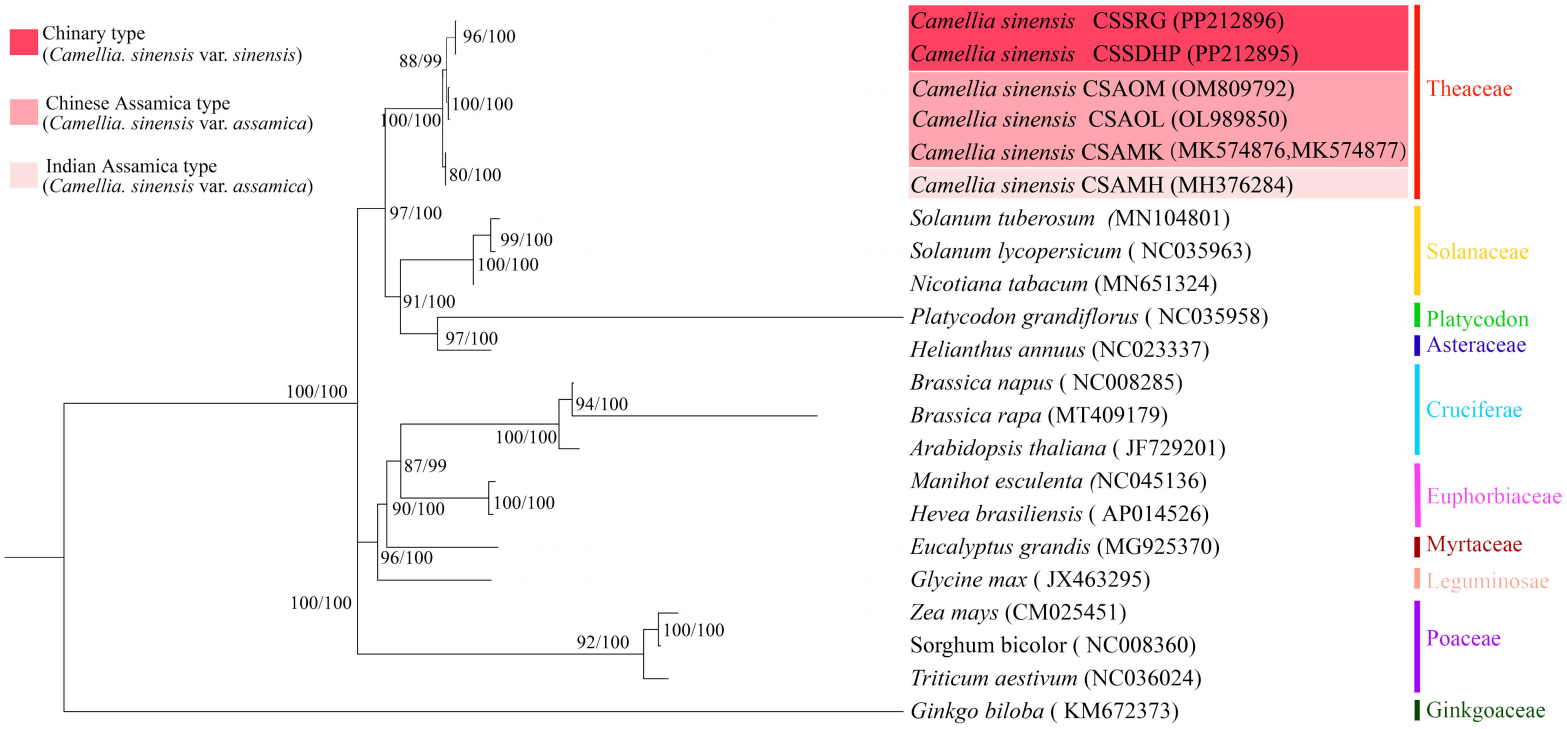
Maximum likelihook (ML) and Bayesian inference (BI) tree based on 20 species. *Zea mays*, *Sorghum bicolor*, *Triticum aestivum*, and *Ginkgo biloba* were classified as outgroups. Numbers beside nodes indicate bootstrap support values and Bayesian posterior probabilities.

To better understand the evolution of *C. sinensis* mitogenome, the phylogenetic trees were generated based on six *C. sinensis* mitogenomes and nineteen other published plant mitogenomes through a combination of the maximum likelihood method and the Bayesian method (**Figure 11**). The results of the classification of phylogenetic trees by the two methods were consistent with each other. Phylogenetic tree results showed that gymnosperms and dicotyledonous plants were different from monocotyledonous plants and dicotyledonous plants respectively, and the clustering of phylogenetic trees matched the family and genus relationships of these species, and Bootstrap analysis showed that all nodes had more than 80% support (BS) and 99% support (BPPs). Among 20 nodes, 17 nodes had a bootstrap value of more than 90% (BS) and BPPs =100%, which confirmed the credibility of the clustering based on mitogenome.

The phylogenetic tree showed that six *C. sinensis* mitogenomes were clustered together and shared a common ancestor. Their ancestors evolved into two clades, one diverging into CSAMK and CSAMH, while the other continued to diverge into CSSDHP and CSSRG, CSAOL and CSAOM, respectively. The topological structure was consistent with collinear cluster (**Figure 2**) and intron cluster results (**Figure 6**).

## 4 Discussion

### 4.1 Comparison of *C. sinensis* mitogenomic characteristics

In order to better understand the evolutionary characteristics of *C. sinensis* mitogenome, two CSS (*C. sinensis* var. *sinensis* cv. Dahongpao, CSSDHP and *C. sinensis* var. *sinensis* cv. Rougui, CSSRG) mitogenomes were high quality gap-free assembled based on a hybrid strategy combining Illumina and Nanopore long sequencing reads, data, and perform comprehensive comparisons with four reported CSA mitogenomes (CSAOL, CSAOM, CSAMK and CSAMH) in terms of their structure, gene content, synteny, intercellular gene transfer, and RNA editing. Mitogenome comparison showed a huge heterogeneity among the six *C. sinensis* species. Complex plant mitogenomes can have circular, branched, linear, or mixed forms of genomic structure (Sloan, 2013; Lai et al., 2022). Of the six genomes, five consisted of a single circular structure, whereas CSAMK mitogenome was unique and consisted of a double circular structure (Zhang et al., 2019). Except for a few transfer RNA (tRNA) genes, the gene content of five mitogenomes was consistent, while CSAMH mitogenome (Rawal et al., 2020) was missing nine genes. The loss of some genes in the mitochondrial genome during evolution had been reported in previous studies (Notsu et al., 2002; Handa, 2003), suggesting that CSAMH might have lost these genes during evolution. However, this could also be due to defects in the early sequencing assembly technology, leading to incomplete assembly. In addition, several genes were found to have copies, the number of copies was not the same in six *C. sinensis* mitogenomes, and the number of introns contained in the genes also varied (**Supplementary Table S2**). In order to better adapt to various environments, multiple copies of functional genes may appear during genome evolution (Liu et al., 2020). Therefore, these multiple copies of functional genes may confer greater stress resistance on tea plants. For example: in this study, compared to the large-leaf tea plant CSAMK, the more cold-tolerant and drought-tolerant small-leaf tea plants CSSDHP and CSSRG have more copies of the Cytochrome coxidase and NADH dehydrogenase genes, which have been reported to be involved in the plant’s defense against stressors such as drought and low temperatures (Liu et al., 2008; Møller et al., 2021; Wang et al., 2022).

Collinearity analysis revealed that six *C. sinensis* mitogenomes had undergone a significant amount of rearrangement, resulting in the order of genes varies greatly, which was consistent with previous studies indicating that there is little conservation of gene order in plant mitogenomes, even among close relatives (Kubo et al., 2000; Satoh et al., 2004). Despite the variability between these mitogenomes, the length of homologous sequences among different plant mitogenomes was consistent with taxonomies, and the closely related species always shared the greatest sequences, even in the non-coding regions (Li et al., 2009). Therefore, these complex mitogenomic DNA structures could also be used to trace common ancestors among diverse species (Xu et al., 2021). In collinear cluster analysis, CSSDHP was clustered with CSSRG, CSAOL was clustered with CSAOM, and CSAMK was clustered with CSAMH, suggesting that CSSDHP and CSSRG, CSAOL and CSAOM, CSAMK and CSAMH had a closer relationship respectively. It was worth mentioning that both CSSDHP and CSSRG come from Fujian Province of China, both CSAOL and CSAOM come from Hunan Province of China, and the place where CSAMK comes from (Yunnan Province of China) and the place where CSAMH comes from (Assam State of India) had similar environmental and climatic characteristics (Willson and Clifford, 1992; Hoffmann et al., 2023). So it also implied that the variation of plant mitogenomic structure might be related to environmental adaptation.

Genomic repetitive sequences had been shown to be essential for intermolecular recombination, which was important evidence of the evolution and genetic characteristics of species (Ammiraju et al., 2007; Hu et al., 2015; Dong et al., 2018). Numerous repetitive sequences, including Long repeat and SSR sequences, were found in six *C. sinensis* mitogenomes, which suggests that frequent intermolecular recombination had dynamically altered the structure and conformation of the mitogenome during the evolution of *C. sinensis.* There were obvious differences in the quantity and types of the repetitive sequence (including Long repeats and SSR) among six *C. sinensis* mitogenomes, which might be caused by gene duplication or variation, as well as geographical and ecological factors (Han et al., 2022). In addition, the number of repetitive sequence in both Long repeat and SSR sequences was found to be proportional to the length of the mitogenome, suggesting that the sequence repetition were closely related to the structure and size of the mitogenome.

During the evolution of plant mitogenome, DNA transfer events have occurred frequently (Timmis et al., 2004). The most common transfer direction is from the plastome to the mitogenome, and the length and sequence similarity of the migrating fragments vary among species (Wang et al., 2012; Zhao et al., 2019). Among four *C. sinensis* studied species, the total length of homologous fragments between chloroplast genome and mitogenomes ranged from 20,733 bp to 21,496 bp, accounting for 1.91% to 2.45% of mitogenomes, respectively. CSSDHP has the largest mitochondrial genome size (1082,025 bp), but homologous fragment with chloroplast genome was not the longest in length (20,733 bp), suggesting that integration of DNA fragments derived from the plastome contributes to limited mitogenome expansion in size. Notably, the total length of repetitive sequences (Long repeats and SSR) accounted for 88.7%- 92.8% of the total length of homologous fragments, suggesting that sequence repetition might be important driver for intracellular gene transfer. In addition, the complete genes detected in the homologous region were all tRNA genes, and tRNA genes were also detected in the homologous with region top-ranked length, which suggested that tRNA genes were more conserved in the mitogenome than protein-coding genes that are transferred, and might be involved in the integration of DNA fragments derived from the plastome. The tRNA genes play a critical role in protein synthesis. Some studies have shown that some tRNA genes in the mitochondrial genome need to be transferred from the plastid genome to maintain the stability and functionality of the mitochondria (Cheng et al., 2021; Yang et al., 2022; Lu et al., 2023).

RNA editing is a post-transcriptional process, which is closely related to the potential molecular functions and physiological processes of mitochondria in higher plants (Sloan and Wu, 2016; He et al., 2018). In this study, the number of predictive RNA editing sites in six *C. sinensis* mitogenome varied from 546 to 720 sites, and both C-to-U and U-to-C types of RNA editing were observed, with more of the C-to-U type. RNA editing that occurs in the first and second positions of codon can lead to changes in the properties of amino acids that affect the function of proteins (Møller et al., 2021). The predictive RNA editing events showed 72.7% of the modifications of the codons altered the amino acids from hydrophilic to hydrophobic, which might contribute to protein stability (Yi et al., 2015). The occurrence of RNA editing would result in a diversity of initiation or termination codons in protein-coding genes (Varre et al., 2019; He et al., 2021; Takenaka, 2022). In six *C. sinensis* mitogenomes, some editing events related to initiation or termination codons also were predicted, including editing event from CGA to UGA in *CcmFC* and *Atp9* genes, from UGA to CGA in *Rps19* gene, and from ACG to ATG in *Cob*, *Cox1*, *Nad5*, *Nad4L* and *Cox2* genes. However, whether these editing events were actually activated at the starting or ending position required further transcriptome experiments to verify. In addition, *Nad4* gene had the most predictive RNA editing sites, followed by *Nad5* gene. Meanwhile, unlike other intron-containing genes, *Nad1*, *Nad2*, and *Nad5* genes have contained not only cis-spliced introns, but also trans-spliced introns, indicating significant editing in the NADH dehydrogenase subunit transcript.

### 4.2 Codon preference and evolutionary characteristics of gene adaptation

During evolution, plant mitogenomes undergo changes in genomic structure and nucleotide composition, as well as loss and transfer of protein-coding genes and tRNA genes, which are thought to be the result of a combination of natural selection, species mutation, and genetic drift (Adams et al., 2002; Liu et al., 2014; Christensen, 2018). Codon use preference play a critical role in protein function, translation accuracy and efficiency, and conducting codon preference analysis could provide insights into these evolutionary fitness of the genome (Hershberg and Petrov, 2008; Tuller et al., 2010).

In this study, the GC content at various positions and the relative synonymous codon usage (RSCU) in six *C. sinensis* mitogenomes were assessed. Total GC content of all six genomes were between 45.62% - 45.75%, indicating a tendency to favor A/T bases, From the RSCU values of codons, a total of 30 codons had RSCU values > 1, of which only one codon was G-ending while the rest were A/T-ending codons, indicating that the genes had little or no bias towards the G/C ending codons in six *C. sinensis* mitogenomes. Further, PR2-plot analysis implied that T (pyrimidines) were used more frequently than A (purines). ENc-plot analysis showed only a few points lay near the curve, and some of the genes with lower ENc values than expected values lay below the curve, which implied that the mutational pressure was not the only factor that contributed to codon use bias, and other factors such as natural selection may play an important role in all six mitogenomes (Kumar et al., 2016). These results were similar to codon preference in the chloroplast genome of *C. sinensis* (Yengkhom et al., 2019), and were consistent with previous studies suggesting that dicot plants exhibit a bias towards A/T-ending codons (Mower, 2020).

In genetics, Ka/Ks ratio is significant for understanding evolutionary dynamics of protein-coding genes across similar and yet diverged species (Xie et al., 2019). A pair Ka/Ks analysis was performed for six *C. sinensis* species, and Ka/Ks ratios of most genes were less than 0.5, indicating that these coding genes were highly conserved and did not undergo rapid evolution during the evolution process (Bi et al., 2022). In contrast, *Nad1* gene and *Sdh3* gene were found to have a Ka/Ks ratio > 1 in pairwise comparisons, suggesting that the two genes had undergone positive selection in *C. sinensis* species. Both *Nad1* and *Sdh3* genes had been reported to be related to plant resistance to stress such as cold tolerance (Fuentes et al., 2011; Sickmann et al., 2011; Møller et al., 2021). CSS is a slower growing shrub with smaller leaves that can withstand cooler climates, while CSA is a fast growing shrub with larger leaves that are highly sensitive to cold weather and mainly grows in warmer tropical regions (Wei et al., 2018). Thus, the positive selection of these two genes might be related to the adaptive evolution of tea plants. Cluster analysis showed that Ka/Ks ratios between the same types (CSS *vs* CSS or CSA *vs* CSA) and between the different types (CSS *vs* CSA) were clearly divided into two clusters, this also further indicated CSS and CSA have undergone different adaptive selections.

### 4.3 Phylogenetic relationship

In this study, the result of phylogenetic tree constructed based on six *C. sinensis* mitogenomes and nineteen other published plant mitogenomes was consistent with the taxonomic information of species. In phylogenetic tree, six *C. sinensis* clustered together, with the CSA and CSS forming two distinct branches. The topological structure was consistent with collinear clustering and intron clustering (**Figures 2**, **10**). This suggested that the CSA and CSS have undergone different evolutionary paths under long-term selective pressures.

Plant mitochondrial gene sequences are highly conserved, so far only a few genes, such as *Atp1*, *Atp9*, *Cob*, *Nad3*, eg., have been reported for phylogenetic relationship analysis (Rawal et al., 2020; Yang et al., 2023). However, due to the insufficient genetic information of a single or a few genes, the resolution of phylogenetic relationship was often unclear. In this study, phylogenetic relationships constructed using complete mitogenome SNPs were consistent with previous phylogenetic relationships based on complete chloroplast genome (Li et al., 2021a) and nuclear genome (Zhang et al., 2021; Cheng et al., 2022; Zhang et al., 2023) with high phylogenetic resolution (BS> 80%, BPPs> 99%). Therefore, it is feasible to construct plant phylogenetic relationships using complete mitogenome SNPs as super molecular markers.

## 5 Conclusion

This is the most detailed comparative description of the sequence, structure, and evolutionary characteristics of *C. sinensis* mitogenomes to date. In this study, two mitogenomes (CSSDHP and CSSRG) were successfully sequenced and assembled, supplementing the information of *C. sinensis* var. *sinensis* (CSS) mitogenome for the first time and comparing them with the *C. sinensis* var. *assamica* (CSA) mitogenomes. The mitogenome of *C. sinensis* exhibited a high degree of heterogeneity in structure, synteny, intercellular gene transfer, and RNA editing, reflecting the outcomes of adaptive evolution. The phylogenetic results are consistent with those of species classification, suggesting the validity of using complete mitogenomes to construct phylogenetic relationships. This study provided an insight into evolution and phylogeny relationship of *C. sinensis* mitogenome, and help to deepen the understanding of the evolution of tea plant.

## Data Availability

The datasets presented in this study can be found in online repositories. The names of the repository/repositories and accession number(s) can be found below: https://www.ncbi.nlm.nih.gov/genbank/, PP212895, https://www.ncbi.nlm.nih.gov/genbank/, PP212896.

## Supplementary material

Supplementary Figure S1 Sequencing Depth and Coverage Map of CSSDHP.

Supplementary Figure S2 Sequencing Depth and Coverage Map of CSSRG.
