# Supplementary figures and images for: Comparative analysis of the complete mitogenomes of Camellia sinensis var. sinensis and C. sinensis var. assamica provide insights into evolution and phylogeny relationship
Source: Front Plant Sci. 2024 Aug 22;15:1396389. doi: 10.3389/fpls.2024.1396389 (PMC11374768; doi:10.3389/fpls.2024.1396389)

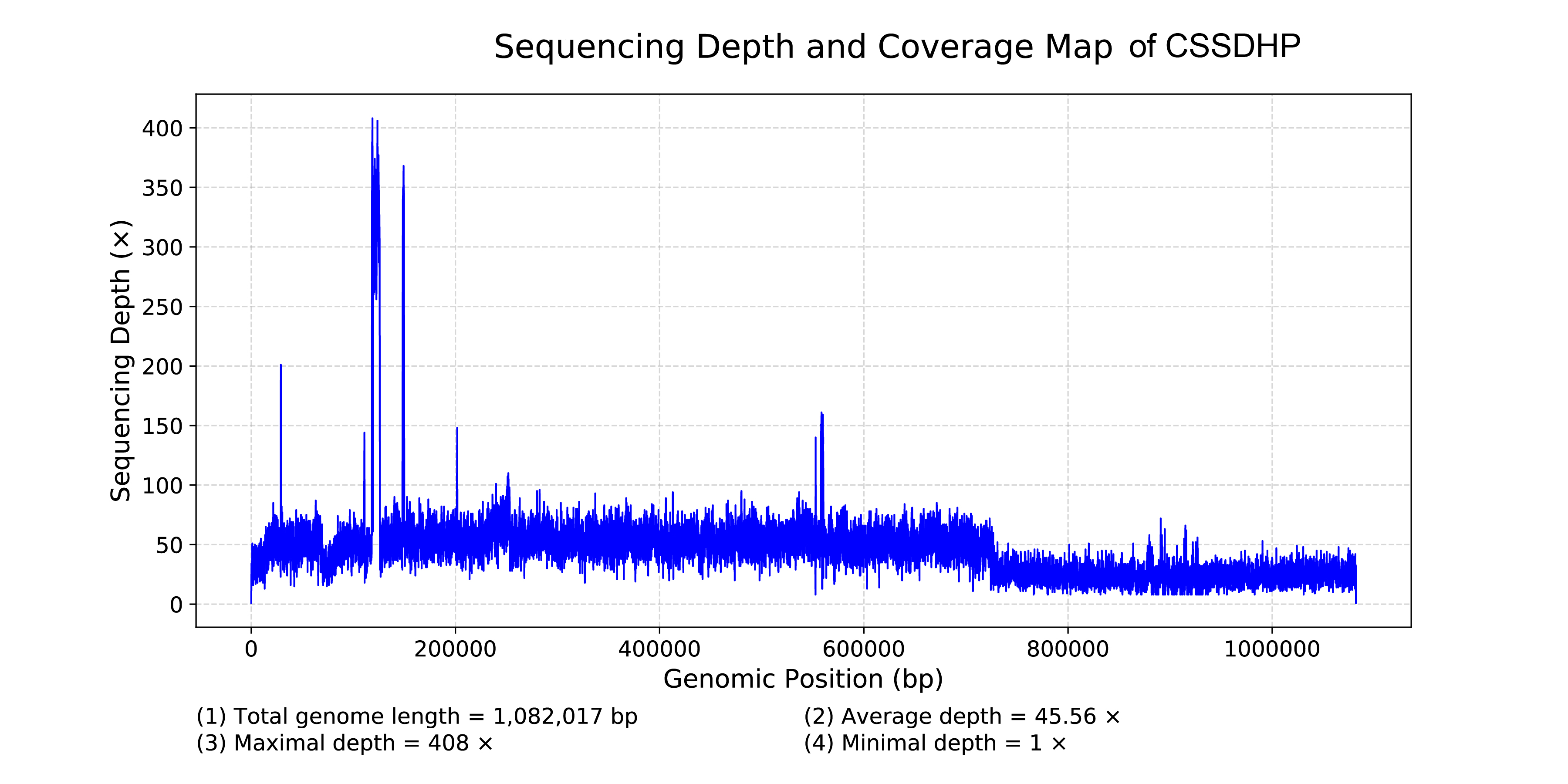

Supplement: Supplementary Figure S1 — Sequencing Depth and Coverage Map of CSSDHP. [file Image1.jpeg]

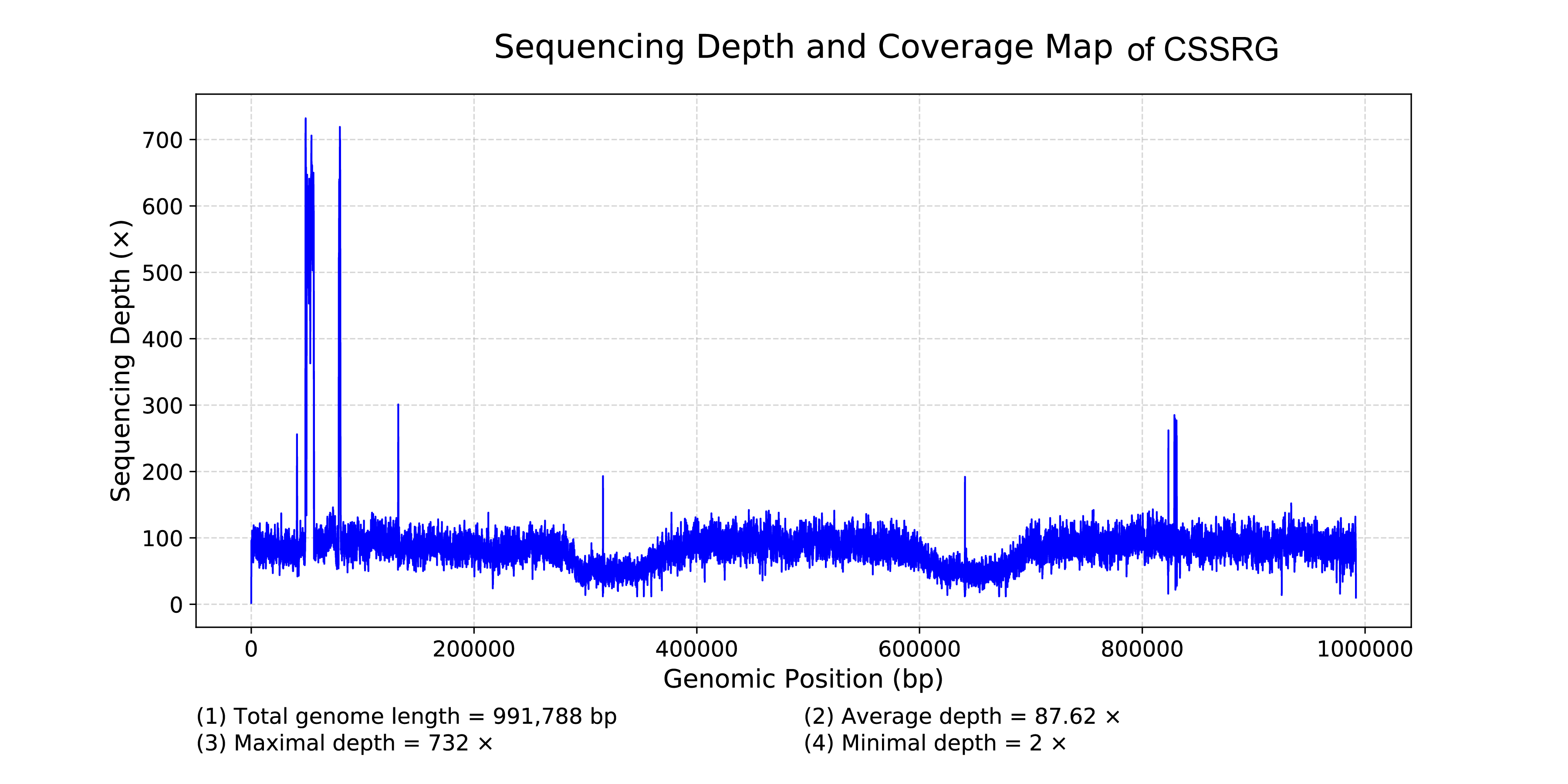

Supplement: Supplementary Figure S2 — Sequencing Depth and Coverage Map of CSSRG. [file Image2.jpeg]
